# Red cell distribution width is associated with stroke severity and unfavorable functional outcomes in ischemic stroke

**DOI:** 10.3389/fneur.2022.938515

**Published:** 2022-11-09

**Authors:** Jie Xue, Dong Zhang, Xiao-Guang Zhang, Xiao-Qiong Zhu, Xu-Shen Xu, Yun-hua Yue

**Affiliations:** Department of Neurology, Yangpu Hospital, Tongji University School of Medicine, Shanghai, China

**Keywords:** red blood cell distribution width (RDW), ischemic stroke, stroke severity, function outcomes, predictor

## Abstract

**Background:**

Red blood cell distribution width (RDW) is considered to be related to coronary heart disease and heart failure and all-cause mortality, but its relationship with acute ischemic stroke is still unclear. In this study, we aimed to explore the relationship between RDW and the stroke severity and functional outcomes of ischemic stroke.

**Methods:**

We retrospectively reviewed patients with acute ischemic stroke between September 2016 and January 2020. Demographic, clinical, stroke complications, laboratory data, and treatment were collected for all patients. Stroke severity and functional outcomes were evaluated by NIHSS score, modified Rankin Scale (mRS), and Barthel Index (BI) at 3 months. Furthermore, multiple logistic regression analysis was used to assess the relationship between RDW and stroke severity and functional outcomes.

**Results:**

A total of 629 patients with acute ischemic stroke were included and were categorized into four groups according to the quartiles of RDW (< 12.4, 12.4–12.9, 13.0–13.4, > 13.4). After multivariable analysis, higher RDW was directly associated with moderate to severe stroke (OR 2.21, 95% CI, 1.30–3.75, *P* = 0.003), mRS score of 3–6 at 3 months (OR 1.86, 95% CI, 1.02–3.41, *P* = 0.044), and BI score below 85 at 3 months (OR 2.27, 95% CI, 1.25–4.12, *P* = 0.007) in patients with ischemic stroke.

**Conclusion:**

Our results demonstrate that RDW is associated with stroke severity and unfavorable functional outcomes at 3 months in patients with ischemic stroke.

## Introduction

Red cell distribution width (RDW) is an indicator reflecting the volume of red blood cells (RBCs) which is routinely calculated during automated cell counters (1). As a measurement method of circulating erythrocyte size variability, it is expressed by the erythrocyte size variation coefficient (2). In previous studies, RDW has been proven to be related to the prognosis of patients with cardiovascular diseases, such as coronary heart disease (3) and heart failure (4, 5), as well as the incidence of all-cause mortality (6, 7).

Acute ischemic stroke is the leading cause of disability and mortality around the globe (8). The annual death rate of stroke in China is about 157 per 100,000 people (9). Given the huge burden of stroke, it is increasingly important to find indicators to evaluate the clinical severity and prognosis of stroke. A large population-based prospective study found an independent relation between higher RDW and the risk of stroke in patients with coronary disease during a median follow-up of 5 years (10). Furthermore, several studies reported that higher RDW was a prognostic factor of poor functional outcome at 3 months (11, 12) and increased mortality (12, 13) in patients with ischemic stroke. In addition, recent studies have shown that in patients with ischemic stroke, higher RDW at baseline is more likely to have poor 1-year prognosis and mortality (14, 15).

There are few reports on the relationship between RDW and stroke severity and functional outcomes in Chinese patients with ischemic stroke. In this study, we aimed to evaluate the association between RDW and stroke severity and functional outcomes in patients with ischemic stroke based on the Chinese population.

## Materials and methods

### Population and study design

This was a retrospective cohort study consisting of patients admitted to the Department of Neurology at Yangpu Hospital Tongji University School of Medicine for acute ischemic stroke between September 2016 and January 2020. The patients who met the following inclusion criteria were included: (1) ≥18 years old; (2) diagnosis of acute ischemic stroke within 7 days of presentation of symptoms; and (3) independent prior to stroke. The exclusion criteria are as follows: (1) infection on admission, (2) a history of hematologic diseases, (3) immune system diseases or use of immunosuppressants, and (4) incomplete clinical and follow-up data. This study was approved by the ethics committee of Yangpu Hospital Tongji University School of Medicine.

### Clinical data

We collected the patients' demographic data (age, gender), clinical characteristics (hypertension, diabetes, atrial fibrillation [AF], coronary heart disease, hyperlipidemia, smoking, and alcohol drinking), stroke complications (post-stroke pneumonia, symptomatic intracranial hemorrhage [sICH]), laboratory parameters, and treatment. Smoking was defined as current or former cigarette smoking. Alcohol consumption was defined as current alcohol intake > 80g/day. Any intracerebral hemorrhage that leads to the deterioration of neurological function is defined as sICH (16). Blood samples were obtained and evaluated in the hospital's biochemistry department within 24 h after admission.

### Stroke severity and function outcomes

The National Institutes of Health Stroke Scale (NIHSS) score was assessed for all patients by qualified neurologists at admission to evaluate stroke severity (17). Based on a previous study, patients were divided into two groups: moderate to severe stroke (≥ 5 points) and mild stroke (< 5 points) (18). We evaluated the prognosis of clinical function by modified Rankin Scale (mRS) and Barthel index (BI) at 3 months after stroke onset. Patients were followed-up by neurologists in our hospital outpatient department or through telephone interviews. Patients with an mRS score of 3–6 at 3 months or with an BI score below 85 at 3 months were considered to have unfavorable functional outcomes (19).

### Statistical analysis

The study population was divided into four groups according to the distribution of RDW in quartiles (Q1–Q4). Continuous variables are expressed as mean ± standard deviation or median of the interquartile range. The Kolmogorov–Smirnov test was used to analyze the normality of distribution. The continuous univariate comparison was performed using the unpaired *t*-test, the Mann–Whitney *U* test, or the Kruskal–Wallis *H* test, when appropriate. Categorical variables are expressed as percentages and analyzed by the chi-square test. Multivariable logistic regression analysis including two models was used to analyze the relationship between RDW and stroke severity and unfavorable functional outcomes, separately. The adjusted variables of model 1 included age, gender, hypertension, diabetes, hyperlipidemia, atrial fibrillation, coronary heart disease; smoking, alcohol drinking, platelet, RBC, WBC, Hs-CRP, FBG, TC, TG, HDL, LDL, the use of antiplatelet, anticoagulation agents, and statin. In addition to the variables of model 1, model 2 added NIHSS score, intravenous thrombolysis, endovascular therapy, symptomatic intracranial hemorrhage, and post-stroke pneumonia as covariates. All statistical analyses with *p*-values < 0.05 were defined as statistically significant. SPSS Statistics 22.0 software (SPSS Inc., Chicago, IL) was performed for all statistical analyses.

## Results

A total of 629 patients with acute ischemic stroke were included in the study. The median age of patients was 70.0 (58.5–73.5) years old, 64.1% of the patients were male subjects. Based on quartiles of RDW, 165 patients were in Q1 (RDW < 12.4), 172 patients were in Q2 (RDW 12.4–12.9), 147 patients were in Q3 (RDW 13.0–13.4), and 145 patients were in Q4 (RDW >13.4). Table 1 shows the baseline characterization of demographics, clinical, stroke complications, laboratory parameters, and treatment according to RDW quartiles. Patients in higher RDW quartiles were older and had a higher prevalence of AF, whereas lower RBC counts were more frequently presented in higher RDW quartiles.

**Table 1:** Baseline characteristics of study patients according to quartiles of RDW.

	**Total (629)**	**RDW**				***P-*value**
		**Q1 (*n* = 165)**	**Q2 (*n* = 172)**	**Q3 (*n* = 147)**	**Q4 (*n* = 145)**	
Age (years)	70.0 (62.0–82.0)	65.0 (58.5–73.5)	70.5 (63.0–81.0)	71.0 (64.0–82.0)	80.0 (65.5–85.0)	<0.001
Gender (male, *n*)	403 (64.1)	112 (67.9)	119 (69.2)	90 (61.2)	82 (56.6)	0.07
Hypertension(*n*)	493 (78.4)	132 (80.0)	134 (77.9)	119 (81.0)	108 (74.5)	0.542
Diabetes (*n*)	209 (33.2)	59 (35.8)	58 (33.7)	49 (33.3)	43 (29.7)	0.722
Hyperlipidemia (*n*)	22 (3.5)	6 (3.6)	7 (4.1)	5 (3.4)	4 (2.8)	0.937
AF (*n*)	80 (12.7)	11 (6.7)	17 (9.9)	21 (14.3)	31 (21.4)	0.001
Coronary heart disease (*n*)	96 (15.3)	17 (10.3)	26 (15.1)	22 (15.0)	31 (21.4)	0.062
Smoking (*n*)	285 (45.3)	86 (52.1)	78 (45.3)	56 (38.1)	65 (44.8)	0.103
Alcohol drinking (*n*)	125 (19.9)	45 (27.3)	32 (18.6)	22 (15.0)	26 (17.9)	0.038
sICH (*n*)	15 (2.4)	3 (1.8)	5 (2.9)	2 (1.4)	5 (3.4)	0.636
Post-stroke pneumonia (*n*)	76 (12.1)	13 (7.9)	20 (11.6)	18 (12.2)	25 (17.2)	0.093
**Laboratory tests**						
RBC (10^12^/L)	4.64 ± 0.60	4.76 ± 0.49	4.66 ± 0.56	4.64 ± 0.60	4.47 ± 0.73	< 0.001
Platelet (10^9^/L)	211.00 (175.00–256.00)	212.00 (172.00–255.00)	219.00 (176.25–254.25)	208.00 (176.00–252.00)	206.00 (168.50–271.00)	0.999
RDW	12.9 (12.3–13.4)	12.1 (11.9–12.2)	12.6 (12.5–12.8)	13.2 (13.1–13.3)	14.1 (13.7–14.7)	
WBC (10^9^/L)	7.10 (5.90–8.90)	6.80 (6.00–9.10)	7.20 (6.00–8.98)	7.60 (6.20–8.90)	7.00 (5.60–9.05)	0.646
**Hs-CRP (mg/L)**						0.109
0 to <5	433 (68.84)	120 (72.73)	122 (70.93)	95 (64.63)	96 (66.21)	
5 to ≤10	94 (14.94)	23 (13.94)	23 (13.37)	31 (21.09)	17 (11.72)	
>10	102 (16.22)	22 (13.33)	27 (15.70)	21 (14.28)	32 (22.07)	
FBG (mmol/L)	7.67 (6.36–10.30)	8.01 (6.51–11.31)	7.49 (6.18–10.46)	8.08 (6.40–10.54)	7.23 (6.25–9.360	0.05
TC (mmol/L)	4.59 (3.84–5.43)	4.71 (3.84–5.39)	4.52 (3.87–5.47)	4.64 (4.05–5.51)	4.50 (3.69–5.31)	0.378
TG (mmol/L)	1.22 (0.86–1.77)	1.39 (1.02–1.91)	1.19 (0.85–1.66)	1.27 (0.85–1.96)	1.07 (0.79–1.56)	<0.001
HDL (mmol/L)	1.09 (0.93–1.27)	1.08 (0.91–1.24)	1.08 (0.89–1.27)	1.14 (0.96–1.32)	1.08 (0.91–1.26)	0.123
LDL (mmol/L)	2.93 (2.43–3.58)	3.02 (2.43–3.59)	2.90 (2.49–3.63)	2.94 (2.46–3.57)	2.86 (2.33–3.54)	0.651
Intravenous thrombolysis (*n*)	170 (27.0)	33 (20.0)	48 (27.9)	38 (25.9)	51 (35.2)	0.027
Endovascular treatment (*n*)	27 (4.3)	3 (1.8)	12 (7.0)	4 (2.7)	8 (5.5)	0.076
Antiplatelet agents (*n*)	594 (94.40)	160 (97.00)	164 (95.30)	137 (93.20)	133 (91.70)	0.191
Anticoagulation agents (*n*)	58 (9.20)	10 (6.10)	15 (8.70)	15 (10.20)	18 (12.40)	0.266
Statin (*n*)	588 (93.50)	158 (95.80)	160 (93.00)	136 (92.50)	134 (92.40)	0.581

AF, atrial fibrillation; FBG, fasting blood glucose; HDL, high-density lipoproteins; Hs-CRP, high sensitive c-reactive protein; LDL, low-density lipoproteins; RBC, red blood cell; RDW, red cell distribution width; sICH, symptomatic intracranial hemorrhage; TC, total cholesterol; TG, triglycerides; WBC, white blood cell; Q1, RDW at first quartiles; Q2, RDW at second quartiles; Q3, RDWat third quartiles; Q4, RDWat fourth quartiles. Data are presented as means (± SD) and medians (IQR) or as a number (percentage).

**Figure 1:**
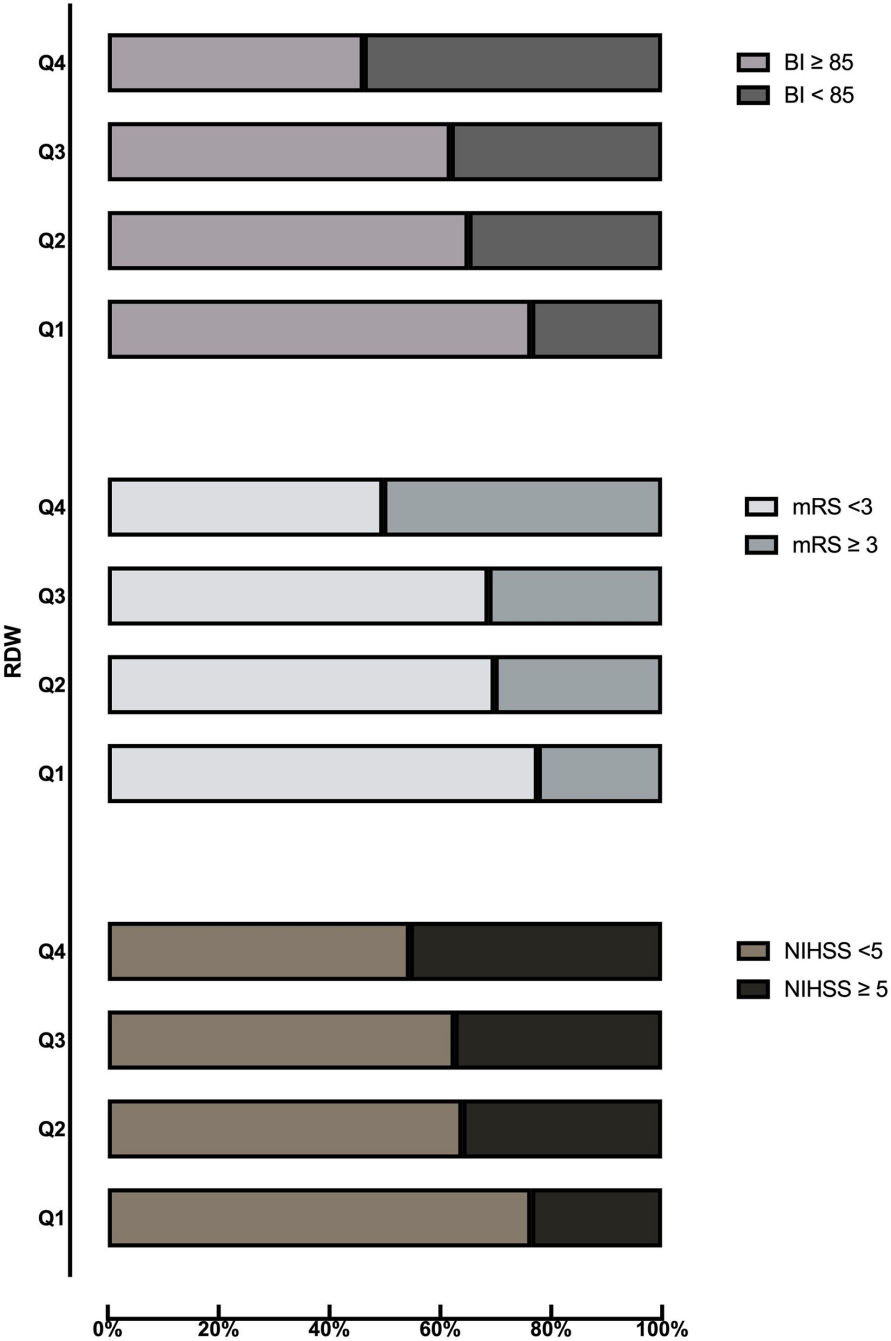
Distribution of NIHSS score, mRS score, and BI score according to quartiles of RDW. BI, Barthel index; mRS, modified Rankin Scale; NIHSS, National Institutes of Health Stroke Scale; RDW, red cell distribution width.

Table 2 shows the comparison of stroke severity and functional outcomes according to quartiles of RDW. Among the 629 patients included, a total of 222 (35.3) had moderate to severe stroke at admission. Patients with higher quartiles had a higher proportion of moderate to severe stroke (*P* = 0.001). At 3 months, there were 208 (33.1) patients with an mRS score of 3 to 6 and 233 (37.0) patients with a BI score below 85. Meanwhile, patients in the Q4 subgroup had a higher frequency of mRS score of 3–6 and BI score below 85 (*P* < 0.001). The distribution of the NIHSS score, mRS score, and BI score according to quartiles of RDW is shown in Figure 1. Table 3 shows the odds ratios for NIHSS, mRS, and BI by RDW quartiles. Univariate analysis demonstrated that patients with RDW in the highest quartile had a higher risk of moderate to severe stroke on admission (OR 2.70, 95% CI, 1.66–4.39, *P* < 0.001) when compared with the first quartile. And patients with RDW in the fourth quartile had a higher relative risk of mRS score of 3 to 6 (OR 3.51, 95% CI, 2.15–5.72, *P* < 0.001) and BI score below 85 (OR 3.76, 95% CI, 2.32–6.11, *P* < 0.001) when compared to the first quartile. After multivariable analysis using model 1 variables, RDW remained associated with moderate to severe stroke (OR 2.21, 95% CI, 1.30–3.75, *P* = 0.003). Further analysis with multiple logistic regression using Model 2 revealed that RDW was associated with mRS score of 3 to 6 (OR 1.86, 95% CI, 1.02–3.41, *P* = 0.044) and BI scores below 85 (OR 2.27, 95% CI, 1.25–4.12, *P* = 0.007).

**Table 2:** Comparison of stroke severity and functional outcomes according to quartiles of RDW.

		**RDW on admission**				
**Outcomes**	**Total (629)**	**Q1 (*n* = 165)**	**Q2 (*n* = 172)**	**Q3 (*n* = 147)**	**Q4 (*n* = 145)**	***P*-value**
Moderate to severe stroke severity (NIHSS ≥ 5)	222 (35.3)	39 (23.6)	62 (36.0)	55 (37.4)	66 (45.5)	0
Unfavorable outcomes at 3 months mRS ≥ 3	208 (33.1)	37 (22.4)	52 (30.2)	46 (31.3)	73 (50.3)	<0.001
BI < 85	233 (37.0)	39 (23.6)	60 (34.9)	56 (38.1)	78 (53.8)	<0.001

BI, Barthel index; mRS, modified Rankin Scale; NIHSS, National Institutes of Health Stroke Scale; RDW, red cell distribution width; Q1, RDW at first quartile; Q2, RDW at second quartiles; Q3, RDWat third quartiles; Q4, RDWat fourth quartiles.

**Table 3:** Risks of stroke severity and functional outcomes according to quartiles of RDW.

	**Univariate analysis**			**Multivariable analysis**	
**Outcomes**	**OR (95% CI)**	***P*-value**		**OR (95% CI)**	***P*-value**
**Moderate to severe stroke severity NIHSS** **≥5**					
Quartile 1	ref			ref	
Quartile 2	1.82 (1.13–2.93)	0.013	Model 1	1.72 (1.05–2.83)	0.031
Quartile 3	1.93 (1.18–3.15)	0.009	Model 1	1.71 (1.01–2.87)	0.044
Quartile 4	2.70 (1.66–4.39)	< 0.001	Model 1	2.21 (1.30–3.75)	0.003
**Unfavorable outcomes at 3 months**					
**mRS** **≥3**					
Quartile 1	ref			ref	
Quartile 2	1.50 (0.92–2.45)	0.105	Model 2	0.89 (0.50–1.60)	0.704
Quartile 3	1.58 (0.95–2.61)	0.078	Model 2	0.82 (0.44–1.52)	0.528
Quartile 4	3.51 (2.15–5.72)	<0.001	Model 2	1.86 (1.02–3.41)	0.044
**BI** **<** **85**					
Quartile 1	ref			ref	
Quartile 2	1.73 (1.07–2.79)	0.024	Model 2	1.18 (0.67–2.07)	0.571
Quartile 3	1.99 (1.22–3.24)	0.006	Model 2	1.30 (0.72–2.33)	0.383
Quartile 4	3.76 (2.32–6.11)	<0.001	Model 2	2.27 (1.25–4.12)	0.007

BI, Barthel index; CI, confidence interval; mRS, modified Rankin Scale; NIHSS, National Institutes of Health Stroke Scale; OR, odd ratio; RDW, red cell distribution width; Q1, RDW at first quartiles; Q2, RDWat second quartiles; Q3, RDWat third quartiles; and Q4, RDWat fourth quartiles. Model 1: Adjusted for age, gender, hypertension, diabetes, hyperlipidemia, atrial fibrillation, coronary heart disease; smoking, alcohol drinking, Platelet, RBC, WBC, Hs-CRP, FBG, TC, TG, HDL, LDL, the use of antiplatelet, anticoagulation agents, and statin. Model 2: Adjusted for Model 1 variables, NIHSS score, intravenous thrombolysis, endovascular treatment, symptomatic intracranial hemorrhage, post-stroke pneumonia.

## Discussion

To our best knowledge, this is the first study to evaluate the association between RDW and stroke severity and functional outcomes in patients with ischemic stroke based on the Chinese population. And we found that RDW was associated with stroke severity and unfavorable functional outcomes at 3 months.

A number of studies have assessed the relationship between RDW and the high risk of stroke in patients with heart diseases. An independent relationship was discovered between elevated RDW and stroke in a study of coronary heart disease patients with a median follow-up of 5 years (10). In another study, increased basal RDW was observed in patients with heart failure having stroke when compared with those without stroke after follow-up for 1 year (20). Moreover, Saliba et al. (21) found that RDW was directly related to stroke risk and improved the prediction accuracy of stroke in patients with AF regardless of anemia status.

In addition, some studies have evaluated the association between RDW and stroke based on the general population. In a case-control study of 224 patients with first ischemic stroke, Ramírez-Moreno et al. (22) found that patients in the fourth quartile of RDW had a significantly higher risk of stroke compared with those in the lower quartile. And during a mean follow-up period of 15.2 years, a large general population study including 26,879 participants reported that RDW in the highest quartile was associated with increased incidence of total stroke and ischemic stroke (23). However, Chen et al. (6) reported that high RDW was independently associated with an increased risk of all-cause mortality, but not the development of stroke in a community population during a median follow-up period of 15.9 years. Furthermore, a series of previous studies have confirmed that RDW is an independent risk factor for increasing poor clinical outcomes at 3 months (11, 12) and all-cause mortality (12, 13, 24, 25) after ischemic stroke. Recently, Ye et al. (26) and Pinho et al. (15) found that baseline RDW is a potential predictor of 1-year survival in patients with ischemic stroke treated with intravenous thrombolysis. Moreover, a retrospective observational single-center study showed that RDW is an independent predictor of 1-year mortality and prognosis in patients with acute anterior circulation stroke after endovascular therapy (14).

Furthermore, there were a few reports on the relationship between RDW and stroke severity, but the conclusions varied. Kara et al. (27) supported that increased RDW values were significantly associated with increased stroke severity in acute ischemic stroke, whereas Ntaios et al. (28) reported that RDW does not predict severity or functional outcome in patients with acute ischemic stroke after multivariable analysis. Our study indicated that the RDW is not only associated with moderate to severe stroke but also associated with unfavorable functional outcomes at 3 months.

Although many studies have shown the relationship between RDW and stroke, its pathophysiological mechanism is not clear. It is widely known that inflammation plays an important role in the pathophysiological processes of ischemic stroke (29, 30). High RDW suggests an increase in extensive RBC size heterogeneity and may reflect a potential inflammatory state, which is associated with adverse clinical outcomes and leads to impaired erythrocyte maturation (10). Previous studies have proved that inflammatory cytokines can inhibit erythropoietin-induced erythrocyte maturation by inhibiting bone marrow, which is reflected by the increase of RDW (31). Inflammatory cytokines and markers such as CRP, interleukin-6, and tumor necrosis factor-alpha have been confirmed to be related to RDW (32–34). Furthermore, high oxidative stress may lead to the increase of RDW by prolonging the survival time of red blood cells (27). And it is reported that the increase in RDW is related to high oxidative stress and low antioxidant level (35, 36). In this study, the relationship between RDW and stroke severity and unfavorable functional outcomes remained significant even after the adjustment of CRP and WBC. Additionally, chronic inflammation and oxidative stress may contribute to the development of atherosclerosis, and the increase of RDW may be an indicator of the development of atherosclerosis (27). Several cohort studies have found a close relationship between high RDW and increased intimal-medial thickness, a well-known risk factor for ischemic stroke (23, 37). Finally, it should be noted that RDW is also related to other medical conditions, including old age, anemia, liver disease, and lung disease, which may affect the clinical prognosis (12).

Several limitations of this study should be acknowledged as follows. First, this was a retrospective observational study with a single center and small sample size. And a few patients were not included in the final analysis due to the lack of clinical or laboratory data, so it might produce selection bias. Second, RDW was only measured once for all participants, so we could not evaluate the RDW dynamically, which is not conducive to a better understanding of the association between RDW and the prognosis of stroke. Finally, we adjusted for possible risk factors in multivariable analysis. However, there may be some residual confounding factors that affect the severity and clinical prognosis of stroke and cannot be completely excluded.

In conclusion, our study demonstrated that RDW is associated with stroke severity and unfavorable functional outcomes at 3 months in patients with ischemic stroke.

## Data availability statement

The raw data supporting the conclusions of this article will be made available by the authors, without undue reservation.

## Ethics statement

The studies involving human participants were reviewed and approved by the Ethics Committee of Yangpu Hospital Tongji University School of Medicine. Written informed consent for participation was not required for this study in accordance with the national legislation and the institutional requirements.

## Author contributions

Y-HY conceived and designed the experiments. X-QZ and X-SX collected the data. DZ and X-GZ analyzed the data. JX wrote the paper. All authors contributed to the article and approved the submitted version.

## Funding

This project was supported by Shanghai Sailing Program (20YF1445000) and the Shanghai Municipal Planning Commission of Science and Research Fund (20204Y0123).

## Conflict of interest

The authors declare that the research was conducted in the absence of any commercial or financial relationships that could be construed as a potential conflict of interest.

## Publisher's note

All claims expressed in this article are solely those of the authors and do not necessarily represent those of their affiliated organizations, or those of the publisher, the editors and the reviewers. Any product that may be evaluated in this article, or claim that may be made by its manufacturer, is not guaranteed or endorsed by the publisher.
